# The Roles of Adipokines, Proinflammatory Cytokines, and Adipose Tissue Macrophages in Obesity-Associated Insulin Resistance in Modest Obesity and Early Metabolic Dysfunction

**DOI:** 10.1371/journal.pone.0154003

**Published:** 2016-04-21

**Authors:** Yea Eun Kang, Ji Min Kim, Kyong Hye Joung, Ju Hee Lee, Bo Ram You, Min Jeong Choi, Min Jeong Ryu, Young Bok Ko, Min A. Lee, Junguee Lee, Bon Jeong Ku, Minho Shong, Ki Hwan Lee, Hyun Jin Kim

**Affiliations:** 1 Department of Internal medicine, Chungnam National University Hospital, Daejeon, Republic of Korea; 2 Department of Obstetrics and Gynecology, Chungnam National University School of Medicine, Daejeon, Republic of Korea; 3 Department of Pathology, Daejeon St. Mary's Hospital, College of Medicine, The Catholic University of Korea, Daejeon, Republic of Korea; 4 Department of internal medicine, Chungnam National University School of Medicine, Daejeon, Republic of Korea; Universidad Pablo de Olavide, Centro Andaluz de Biología del Desarrollo-CSIC, SPAIN

## Abstract

The roles of adipokines, proinflammatory cytokines, and adipose tissue macrophages in obesity-associated insulin resistance have been explored in both animal and human studies. However, our current understanding of obesity-associated insulin resistance relies on studies of artificial metabolic extremes. The purpose of this study was to explore the roles of adipokines, proinflammatory cytokines, and adipose tissue macrophages in human patients with modest obesity and early metabolic dysfunction. We obtained omental adipose tissue and fasting blood samples from 51 females undergoing gynecologic surgery. We investigated serum concentrations of proinflammatory cytokines and adipokines as well as the mRNA expression of proinflammatory and macrophage phenotype markers in visceral adipose tissue using ELISA and quantitative RT-PCR. We measured adipose tissue inflammation and macrophage infiltration using immunohistochemical analysis. Serum levels of adiponectin and leptin were significantly correlated with HOMA-IR and body mass index. The levels of expression of MCP-1 and TNF-α in visceral adipose tissue were also higher in the obese group (body mass index ≥ 25). The expression of mRNA MCP-1 in visceral adipose tissue was positively correlated with body mass index (r = 0.428, p = 0.037) but not with HOMA-IR, whereas TNF-α in visceral adipose tissue was correlated with HOMA-IR (r = 0.462, p = 0.035) but not with body mass index. There was no obvious change in macrophage phenotype or macrophage infiltration in patients with modest obesity or early metabolic dysfunction. Expression of mRNA CD163/CD68 was significantly related to mitochondrial-associated genes and serum inflammatory cytokine levels of resistin and leptin. These results suggest that changes in the production of inflammatory biomolecules precede increased immune cell infiltration and induction of a macrophage phenotype switch in visceral adipose tissue. Furthermore, serum resistin and leptin have specific roles in the regulation of adipose tissue macrophages in patients with modest obesity or early metabolic dysfunction.

## Introduction

Adipose tissue is considered an endocrine organ that secretes various adipokines involved in metabolic regulation and inflammatory processes [1]. Dysregulation of endocrine function and inflammation of adipose tissue (AT) induce low-grade systemic inflammation and insulin resistance in obese patients, which are implicated in the pathophysiology of type 2 diabetes mellitus (T2DM), atherosclerosis, hypertension, and metabolic syndrome [2–4]. Research on the link between obesity and insulin resistance has focused on adipokines and AT inflammation. Adipokines contribute to the development of obesity-associated insulin resistance and have a paracrine and autocrine role in crosstalk among diverse cell types, including endothelial cells, fibroblasts, and various immune cells in the AT [5–7]. Macrophages are reportedly the key immune cells responsible for AT inflammation in obese patients [8, 9]. During a chronic inflammatory state, the accumulation of macrophages in AT induces impaired adipocyte functioning and may contribute to the development of insulin resistance [10, 11]. Moreover, macrophages have a significant role in the link between the innate and adaptive immune system [8] and are another source of inflammatory cytokines associated with insulin resistance.[12] Additionally, obesity induces a phenotypic switch in macrophage polarization and increases proinflammatory cytokines (TNF-α, IL1β, MCP-1). These cytokines are produced primarily from macrophages, which increase to account for 40% of total cells in AT [8, 12].

Our current mechanistic understanding of obesity-associated AT inflammation and insulin resistance is based primarily on animal studies of artificial metabolic extremes (high-fat diet challenges, lipid infusion, or genetic intervention) or human studies of severely obese subjects [13–15]. These studies provide important insights, but their results must be validated by human studies that are designed to examine the non-extreme forms of obesity-induced metabolic disease that dominate the clinical landscape. Several studies have examined the role of adipokines in patients with non-severe obesity, but less is known about the role of adipose tissue macrophages (ATMs) in non-severe obesity-associated insulin resistance [16, 17]. This is an important field of obesity-related research, because pharmacological approaches that target the molecules that contribute to insulin resistance in modest obesity and early metabolic dysfunction are essential. These approaches can prevent the development of chronic obesity-induced metabolic dysfunctions such as cardiovascular disease, diabetes mellitus, and metabolic syndrome.

In this study, we investigated adipokines, proinflammatory cytokines, AT inflammation and ATMs markers to clarify the relationship among these factors and to determine the role of adipokines, inflammation, and the macrophage phenotype in obesity-associated insulin resistance among patients with modest obesity and early metabolic dysfunction.

## Materials and Methods

### Patients and tissue samples

In total, 62 women were recruited for the study. These women all underwent an electric gynecologic operation at the Center for Gynecology, a unit of the Chungnam National University Hospital. Reasons for surgery varied and included one or more of the following conditions: myoma, adenomyomatosis, ovarian mucinous cystadenoma, ovarian serous cystadenoma, ovarian teratoma, adenomatous hyperplasia, cervical squamous cell carcinoma, hydrosalpinx, and ovarian cysts. Inclusion criteria for the study were absence of any clinical sign of infection or inflammation, systolic blood pressure less than 140 mmHg and diastolic blood pressure less than 100 mmHg, no alcohol or drug abuse, and no pregnancy. Of the 62 candidates, 11 did not meet the inclusion criteria resulting in 51 patients being included. The Institutional Review Board of Chungnam National University Hospital approved the protocol for this research, and written informed consent was obtained from all participants. The experimental protocol was performed in accordance with the Declaration of Helsinki.

### Definition of obesity, T2DM, and prediabetes

We defined obesity as a body mass index (BMI) of at least 25. This was calculated as weight in kilograms divided by height in meters squared, following the WHO Asia–Pacific classification guidelines [18]. T2DM mellitus was defined as medical treatment for diabetes, a fasting blood glucose (FBG) level of 126 mg/dL or greater, a 2-h blood glucose level of 200 mg/dL or greater during an oral glucose tolerance test (OGTT), a non-fasting blood glucose level of 200 mg/dL or greater with classic hyperglycemic symptoms, or glycated hemoglobin (HbA1c) levels of 6.5% or greater. Prediabetes was defined using either impaired fasting glucose (IFG) levels with an FBG level of 100–125 mg/dL or impaired glucose tolerance (IGT) with a 2-h blood glucose level during an OGTT of 140–199 mg/dL or HbA1c levels of 5.7–6.4% [19].

### Body composition measurements

The degrees of overall obesity and abdominal obesity were assessed using height and waist circumference. Height and body weight were measured using a digital scale while the subject wore a light gown. Waist circumference was measured with a tape measure to the nearest 0.1 cm at the midpoint between the lower costal margin and the iliac crest. Total body-fat-mass and total muscle-mass percentages were assessed using an eight-polar tactile-electrode impedance-meter (Inbody 770, Seoul, Korea).

### Blood chemistry and lipid profiles

Venous blood samples and an OGTT were taken after at least a 12-h fast on the morning of surgery or during the days preceding surgery. Blood chemistry and lipid profiles were measured using a blood chemistry analyzer (Hitachi 747; Hitachi, Tokyo, Japan). HbA1c levels were measured using high-performance liquid chromatography (Bio-Rad, Hercules, CA, USA). Inulin and C-peptide levels were measured using radioimmunoassays (Roche, Penzberg, Germany). We calculated insulin resistance using the homeostasis model assessment insulin resistance (HOMA-IR): [fasting insulin (mU/mL) X fasting glucose (mmol/L)] / 22.5. We calculated insulin secretory capacity using the homeostasis model assessment beta cell function (HOMA-β): [(20 X fasting insulin (mU/mL) / (fasting plasma glucose– 3.5)].

### ELISA

The fasting serum adiponectin, leptin, resistin, IL 1β, MCP-1, and TNF-α levels were measured using a quantitative sandwich enzyme immunoassay technique and an enzyme-linked immunosorbent assay (Adiponectin: ELISA, R&D systems, Minneapolis, MN, USA, catalog number HADK1MAG-61K; Leptin: ELISA, R&D systems, Minneapolis, MN, USA, catalog number HADK2MAG-61K; Resistin: ELISA, R&D systems, Minneapolis, MN, USA, catalog number HADK1MAG-61K; IL 1β: ELISA, R&D systems, Minneapolis, MN, USA, catalog number HADK2MAG-61K; MCP-1: ELISA, R&D systems, Minneapolis, MN, USA, catalog number HADK2MAG-61K; and TNF-α: ELISA, R&D systems, Minneapolis, MN, USA, catalog number HADK2MAG-61K).

### AT sampling and adipocyte fraction

We collected omental AT samples from the distal portion of the greater omentum. Biopsy specimens were placed in an ice-cold saline solution and transported to the laboratory within 15 min of collection. Half portions of fresh AT samples were fixed in 10% neutralized formalin for 24 h, followed by tissue processing for 10 h and then embedding in paraffin wax. To isolate adipocyte, non-frozen tissues were washed three to four times with phosphate-buffered saline (PBS) and suspended in an equal volume of PBS supplemented with 1% penicillin–streptomycin and 0.1% collagenase type I, pre-warmed to 37°C. The tissue was placed in a shaking water bath at 37°C with continuous agitation for 60 min, followed by centrifugation at room temperature for 5 min at 300 g to 500 g. The supernatants, which contained mature adipocytes, were re-collected and incubated in a gas phase of 95% O_2_ and 5% CO_2_.

### Histological studies

Paraffin-embedded tissue sections (4 μm thick) were placed in an oven and incubated at 56°C for 3 h prior to hematoxylin and eosin (H&E) and immunohistochemical stainings. All procedures, including antigen retrieval and blocking of endogenous peroxidase activity, were performed automatically using the BenchMark HX automated system (Ventana Medical Systems, SA, Illkirch Cedex, France). Tissue sections were incubated with an anti-CD68 primary antibody (Bioss Inc.) for 32 min at 42°C. Immunoperoxidase staining was performed using the LSAB system NeuVision (Ventana), according to the manufacturer’s instructions, and counterstained with hematoxylin. Tissue slides were analyzed using an OLYMPUS BX51 microscope.

A certified pathologist (J Lee) performed the microscopic analysis and was unaware of the identity of the samples. Macrophages, defined as CD68-immunostained cells, and crown-like structures, defined as an adipocyte completely surrounded by macrophages, were counted in high-power fields (HPFs) at a magnification of X 400. We further calculated the numbers of macrophage foci and crown-like structures per 10 HPFs. For the analysis of inflammation status in visceral adipose tissue (VAT), the degree of inflammation (neutrophil and lymphocyte infiltrates) was graded as mild (grade 1), moderate (grade 2), or severe (grade 3).

### Messenger RNA isolation and quantitative real-time reverse transcriptase polymerase chain reaction (RT-PCR)

Total RNA was isolated using Trizol (Invitrogen, Life Technologies, Carlsbad, CA, USA). Complementary DNA (cDNA) was prepared from the total RNA using M-MLV Reverse Transcriptase and oligo-dT primers (Invitrogen). Real-time PCR was performed using cDNA, QuantiTect SYBR Green PCR Master Mix (QIAGEN), and specific primers (S1 Table). The PCR reactions were performed followed by 40 PCR cycles, with each cycle performed at 95°C for 15 sec, 60°C for 1 min, and 72°C for 1 min.

### Statistical analysis

All data are expressed as means ± SDs. We compared clinical characteristics, biochemical data, and inflammatory molecules between the lean group and the obese group using unpaired *t*-tests for parametric data and Mann–Whitney U-tests for nonparametric data. One-way analysis of variance was used to compare parameters among the normal-glucose-tolerance, prediabetes, and diabetes groups. To analyze the strength of the relationship between variables of interest, Spearman correlation coefficients were used. Statistical significance was set at *P* ≤ 0.05 and *P* ≤ 0.01. Statistical analyses were performed using SPSS version 20 (IBM Co., Armonk, NY, USA).

## Results

### Characteristics of study population

In total, 51 participants (mean age 46.55 years ± 6.40 years, 100% female) completed the study. The mean BMI, waist circumference (WC), waist-to-hip ratio (WHR), and HOMA-IR values were 24.27 kg/m^2^ ± 2.97 kg/m^2^, 81.8 cm ± 7.43 cm, 0.885 ± 0.052, and 2.329 ± 1.355, respectively. These figures show that the study population was relatively non-severely obese and non-severely hyperglycemic. The clinical characteristics of the study participants stratified by BMI (< 25 vs. ≥ 25) are shown in Table 1. The BMI, waist circumference, body fat portion, body fat mass, and HOMA-IR were all significantly higher in the obese group than in the lean group, whereas the groups did not differ significantly with regard to the other parameters (Table 1). In terms of glucose tolerance, we found significant differences among groups (normal glucose tolerance vs. prediabetes vs. diabetes) in the mean body-fat mass and fasting glucose, post-load 2-h glucose, HbA1c, HOMA-IR, and HOMA-β levels, as expected (S2 Table).

**Table 1:** Characteristics of study population by BMI.

Variable	Lean Group (BMI < 25) (N = 29)	Obese Group (BMI ≥ 25) (N = 22)	*P*-value^a^
**Age (years)**	47.83 ± 5.18	44.86 ± 7.52	0.102
**BMI (kg/m**^**2**^**)**	22.37 ± 1.65	26.80 ± 2.39	0.000
**Waist circumference (cm)**	78.27 ± 4.93	86.46 ± 7.70	0.000
**Body fat (%)**	27.63 ± 3.91	34.16 ± 4.29	0.000
**Body fat mass (kg)**	16.36 ± 4.81	22.88 ± 5.23	0.000
**Muscle mass (kg)**	37.43 ± 5.20	55.15 ± 71.43	0.259
**Fasting glucose (mmol/L)**	5.47 ± 1.06	6.26 ± 2.33	0.149
**Fasting insulin (pmol/L)**	56.19 ± 24.03	68.76 ± 25.28	0.077
**Post-load 2 h glucose (mmol/L)**	8.73 ± 4.65	10.38 ± 5.28	0.245
**Post-load 2 h insulin (pmol/L)**	360.24 ± 287.45	449.90 ± 336.42	0.316
**HbA1c (%)**	5.44 ± 0.98	5.93 ± 1.36	0.142
**HOMA-IR**	1.98 ± 0.96	2.79 ± 1.66	0.032
**HOMA-β**	92.74 ± 47.15	96.65 ± 46.52	0.769
**TG (mmol/L)**	1.24 ± 0.89	1.28 ± 0.73	0.872
**LDL-chol (mmol/L)**	2.90 ± 0.94	3.03 ± 0.76	0.607

BMI, body mass index; HbA1c, glycated hemoglobin; TG, triglycerides; LDL, low density lipoprotein. Data are presented as means ± SDs.

^a^*p-*value from unpaired *t-*test for continuous parametric variables and Mann–Whitney U-test for nonparametric variables.

### Differences in proinflammatory markers and macrophage markers by obesity level

**Fig 1:**
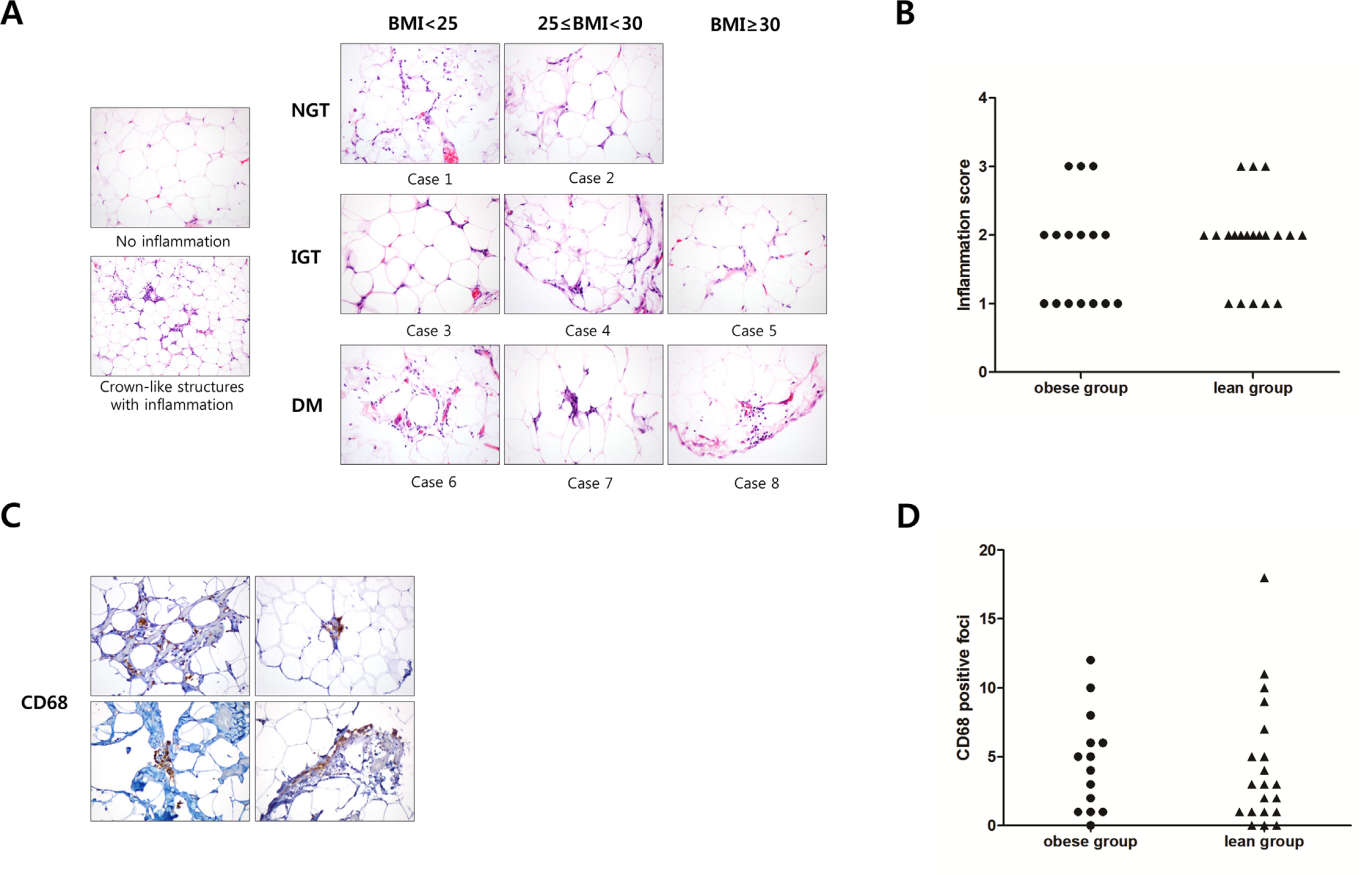
Inflammation and CD68+ macrophage infiltration in human visceral adipose tissue. (A) The adipose tissue inflammation of the groups did not differ in relation to glucose tolerance and BMI. (B) The inflammatory scores of the adipose tissue of the lean and the obese groups did not differ. (C) The CD68-positive foci were examined. The pattern was heteregenously individualized. (D) The number of CD68-positive foci of the lean and obese groups did not differ.

Table 2 shows serum concentrations of adipokines and proinflammatory markers, and mRNA expression levels of proinflammatory markers and macrophage markers in VAT by obesity level. Leptin concentrations were higher and adiponectin levels were lower in the obese group than in the lean group. There were no differences in the serum levels of resistin and proinflammatory markers. However, the mRNA expressions of MCP-1 and TNF-α in VAT were significantly higher in the obese group than in the lean group. There was no difference in the expression of the macrophage markers, including CD68, CD163/CD68, and CD206/CD68, between the two groups. We analyzed the differences in relation to glucose tolerance, but there were no significant differences in proinflammatory markers or macrophage markers in serum or VAT (S3 Table). The general macrophage marker (CD68) and inflammatory scoring were analyzed by immunohistochemical staining in VAT from some subjects (n = 36) (Fig 1). Crown-like structures with inflammation were observed in some participants, but there was no difference between the obese group and the lean group (Fig 1). The number of CD68+ foci and the inflammatory scoring in VAT showed no difference between the obese and lean groups (Fig 1). We analyzed the relationship between the inflammatory score and BMI, glucose tolerance, adipokines, and proinflammatory biomarkers, but no significant relationship was observed (data not shown).

**Table 2:** Adipokine, proinflammatory, and macrophage markers by obesity level.

Adipokine, Proinflammatory, and macrophage markers	Lean Group (BMI < 25) (N = 29)	Obese Group (BMI ≥ 25) (N = 22)	*P*-value^a^
** Serum Adiponectin (μg/mL)**	15.07 ± 13.88	8.08 ± 5.61	0.026
** Serum Leptin (ng/mL)**	6.96 ± 4.49	12.69 ± 8.70	0.008
** Serum Resistin (ng/mL)**	54.96 ± 34.41	51.88 ± 29.20	0.846
** Serum IL-1β (pg/mL)**	3.81 ± 8.92	1.69 ± 2.15	0.282
** Serum MCP-1 (pg/mL)**	192.23 ± 90.87	209.72 ± 94.76	0.531
** Serum TNF-α (pg/mL)**	4.30 ± 2.57	4.76 ± 3.49	0.596
** Serum hs CRP (mg/L)**	0.83 ± 0.77	0.83 ± 0.60	0.999
** VAT Adiponectin**	0.44 ± 0.42	0.88 ± 2.20	0.539
** VAT MCP-1**	5.10 ± 8.87	16.83 ± 16.41	0.007
** VAT TNF-α**	12.77 ± 13.98	501.73 ± 752.31	0.007
** VAT CD68**	37.30 ± 168.12	9.80 ± 14.06	0.114
** VAT CD163/CD68**	7.48 ± 11.86	5.04 ± 6.12	0.844
** VAT CD206/CD68**	11.96 ± 21.06	9.09 ± 22.49	0.417

IL-1β, interleukin-1 beta; MCP-1, monocyte chemoattractant protein-1; TNF-α, tumor necrosis factor-α; hs CRP, high sensitivity C-reactive protein; VAT, visceral adipose tissue. Data are presented as means ± SDs.

^a^*p-*value from unpaired *t*-test for continuous parametric variables and Mann–Whitney U-test for nonparametric variables.

### Correlation of proinflammatory markers with obesity and insulin resistance

The correlations of BMI and HOMA-IR with adipokines and proinflammatory markers are shown in Table 3. Serum adiponectin was negatively correlated with BMI (r = -0.415, *p* = 0.002) and HOMA-IR (r = -0.598, *p* < 0.001). Serum leptin was also significantly correlated with BMI (r = 0.625, *p* < 0.001) and HOMA-IR (r = 0.362, *p* = 0.009). Expression of mRNA MCP-1 in VAT was positively correlated with BMI (r = 0.428, *p* = 0.037) but not with HOMA-IR (r = 0.122, *p* = 0.571). However, TNF-αin VAT was not correlated with BMI (r = 0.323, *p* = 0.153), but was correlated with HOMA-IR (r = 0.462, *p* = 0.035).

**Table 3:** Correlation of proinflammatory markers with obesity and insulin resistance.

Factors	BMI	HOMA-IR
**Serum Adiponectin**	-0.415**	-0.598**
**Serum Leptin**	0.625**	0.362**
**Serum Resistin**	-0.048	0.099
**Serum MCP-1**	0.049	0.000
**Serum TNF-α**	0.034	0.103
**VAT MCP-1**	0.428*	0.122
**VAT TNF- α**	0.323	0.462*
**VAT CD68**	0.247	-0.142

BMI, body mass index; MCP-1, monocyte chemoattractant protein-1; TNF-α, tumor necrosis factor-α; VAT, visceral adipose tissue. Data are presented as Spearman’s *R* values

* *p* < 0.01

** *p* < 0.05.

### Differences in proinflammatory markers and macrophage phenotypes in relation to insulin resistance in the obese group

To identify the major factors contributing to obesity-associated insulin resistance, we analyzed the proinflammatory markers and macrophage markers in serum and VAT in relation to HOMA-IR in the obese group (Table 4). Only serum adiponectin levels were significantly lower in the insulin-resistant group (HOMA-IR ≥ 2.5) than in the normal insulin-sensitive group (HOMA-IR < 2.5). However, there was no difference in other adipokines or in proinflammatory or macrophage markers, including the mRNA expression of MCP-1 and TNF-α, which reflected significant differences between the lean group and the obese group in the total sample.

**Table 4:** Metabolic parameters of the obese group by HOMA-IR.

Metabolic parameters	HOMA-IR < 2.5 (N = 11)	HOMA-IR ≥ 2.5 (N = 11)	*P*-value^a^
**Fasting glucose (mmol/L)**	5.31 ± 0.84	7.21 ± 2.95	0.053
**HbA1c (%)**	5.25 ± 0.46	6.61 ± 1.63	0.015
**Fasting insulin (pmol/L)**	50.62 ± 12.22	86.81 ± 21.60	0.000
**TG (mmol/L)**	0.99 ± 0.46	1.58 ± 0.85	0.057
**HOMA-IR**	1.70 ± 0.37	3.88 ± 1.73	0.001
**Serum Adiponectin (pg/mL)**	11.33 ± 5.95	4.83 ± 2.74	0.004
**Serum Resistin (pg/mL)**	51.85 ± 33.59	51.90 ± 25.72	0.996
**Serum IL-1β (pg/mL)**	1.91 ± 1.82	1.47 ± 2.51	0.644
**Serum Leptin (pg/mL)**	9.76 ± 4.88	15.62 ± 10.79	0.116
**Serum MCP-1 (pg/mL)**	192.22 ± 99.35	226.23 ± 91.17	0.399
**Serum TNF-α (pg/mL)**	3.44 ± 2.50	6.08 ± 3.93	0.077
**Serum hs CRP (mg/L)**	0.77 ± 0.45	0.87 ± 0.71	0.708
**VAT Adiponectin**	0.24 ± 0.22	1.52 ± 3.15	0.357
**VAT MCP-1**	19.20 ± 15.38	14.47 ± 18.50	0.392
**VAT TNF- α**	845.14 ± 892.06	72.48 ± 77.56	0.071
**VAT CD68**	11.67 ± 13.31	7.94 ± 15.17	0.865
**VAT CD206/CD68**	13.82 ± 30.18	3.84 ± 7.32	0.547

HbA1c, glycated hemoglobin; TG, triglycerides; IL-1β, interleukin-1 beta; MCP-1, monocyte chemoattractant protein-1; TNF-α, tumor necrosis factor-α; hs CRP, high sensitivity C-reactive protein; VAT, visceral adipose tissue. Data are presented as means ± SDs.

^a^*p*-value from unpaired *t*-test for continuous parametric variables and Mann–Whitney U-test for nonparametric variables.

### Correlation of macrophage markers with metabolic parameters and proinflammatory and mitochondria-related markers

We evaluated which factors can determine the macrophage phenotype. Table 5 shows the correlations between metabolic parameters and the genetic expression of macrophage markers, including the total (CD68) and M2 macrophage proportions (CD163/CD68, CD206/CD68). Serum leptin and resistin were correlated with CD163/CD68 (r = 0.452, *p* = 0.001). Interestingly, the expression of genes from mitochondrial biogenesis and the OXPHOS complex, such as PGC 1α, PGC 1β, and NDUFA from the adipocytes, was significantly positively correlated with CD163/CD68 (PGC 1α, r = 0.504, *p* < 0.001; PGC 1β, r = 0.326, *p* = 0.029; and NDUFA, r = 0.424, *p* = 0.003) (Table 5).

**Table 5:** Correlation coefficients of macrophage markers for all participants.

Factors	CD68	CD206/CD68	CD163/CD68
**BMI**	0.247	-0.134	-0.085
**WC**	0.293*	-0.138	-0.062
**HOMA-IR**	-0.142	-0.141	-0.066
**Serum Adiponectin**	-0.0.080	0.164	0.077
**Serum Leptin**	0.129	-0.097	-0.291**
**Serum Resistin**	0.103	-0.073	0.452**
**Serum IL-1β**	0.046	0.047	-0.126
**Serum MCP-1**	0.265	-0.230	-0.175
**Serum TNF-α**	0.077	-0.206	-0.037
**Serum hs CRP**	0.062	-0.020	-0.060
**VAT adiponectin**	-0.160	-0.062	0.178
**VAT MCP-1**	-0.062	0.077	-0.188
**VAT TNF- α**	0.090	-0.221	-0.242
**Adipocyte PGC1α**	0.164	-0.321*	0.504**
**Adipocyte PGC1 β**	-0.166	0.115	0.326**
**Adipocyte NDUFA**	0.015	-0.066	0.424**

BMI, body mass index; WC, waist circumference; IL-1β, interleukin-1 beta; MCP-1, monocyte chemoattractant protein-1; TNF-α, tumor necrosis factor-α; hs CRP, high sensitivity C-reactive protein; VAT, visceral adipose tissue; PGC1α, peroxisome proliferator-activated receptor gamma coactivator 1-alpha; PGC1 β, peroxisome proliferator-activated receptor gamma coactivator 1-beta; NDUFA, NADH dehydrogenase 1 alpha subcomplex subunit. Data are presented as Spearman’s *R* values.

* represents statistically significant correlation where the *p-*value < 0.01, and

** represents a statistically significant correlation where the *p*-value < 0.05.

## Discussion

To our knowledge, this is the first study to demonstrate the relationship among adipokines, ATMs, and proinflammatory markers in human patients with modest obesity and early metabolic dysfunction. There were no differences in the degree of inflammation or macrophage marker expression in the AT between groups stratified according to levels of obesity and insulin resistance. However, circulating levels and tissue-expression levels of adipokines and proinflammatory markers changed significantly in the obese group. These results suggest that changes in the production of inflammatory biomolecules precede increased inflammation or switch off the macrophage phenotype of AT in obesity-associated insulin resistance. In this study, the mean BMIs in the lean group and the obese group according to the WHO Asia–Pacific classification for obesity were 22.37 kg/m^2^ and 26.80 kg/m^2^, respectively^2^. Despite the slight difference in BMI, there were significant differences in metabolic parameters, including body-fat mass, body-fat percentage, and insulin resistance, as represented by HOMA-IR. Compared with other human studies on AT inflammation and macrophages, the narrow range of BMIs in this study is more reflective of the clinical landscape, and the use of this population is more likely to yield information about the major contributing factors to obesity-associated insulin resistance in patients with modest obesity and early metabolic dysfunction.

### The roles of adiponectin and leptin in the obesity-associated insulin resistance of individuals with modest obesity

Previous studies have reported decreased serum levels of adiponectin in obese subjects compared with non-obese subjects as well as negative correlations between adiponectin and BMI and negative associations among plasma adiponectin levels, insulin sensitivity, and anti-inflammatory activity in rodent models and humans [20–22]. Adiponectin gene expression is lower in VAT than in subcutaneous adipose tissue (SAT), and SAT might be more important for circulating adiponectin levels [23]. In this study, serum adiponectin levels decreased and levels of adiponectin were correlated with BMI and HOMR-IR. However, there was no direct correlation between adiponectin gene expression and serum adiponectin levels, and no relationship was found between genetic expression and any of the factors relevant to insulin resistance. The discrepancy in these results may be due to the specific study subjects with modest obesity and early metabolic dysfunction and/or the fact that the AT studies have been performed only on omental AT after adipocyte isolation. Importantly, we documented a significant decrease in serum adiponectin levels in the insulin-resistant group, but there was no significant difference in other adipokines or proinflammatory cytokines. Our study supports the hypothesis that adiponectin may be the strongest and earliest key molecule in obesity-associated insulin resistance in patients with modest obesity when other adipokines and proinflammatory cytokines do not change and inflammation does not yet dominate. Previous studies observed an inverse relationship between insulin resistance and adiponectin mRNA expression and demonstrated an association between low levels of adiponectin and diverse inflammatory mediators [24, 25]. Therefore, the insulin resistance observed here may represent a consequence, rather than a cause, of the decreased adiponectin expression in adipose tissue. In post-menopausal women, changes in estrogen bioactivity and reduced circulating adiponectin levels have been observed; however, we were unable to measure the effect of menopausal status or to adjust for menopausal status. Therefore, this represents a limitation of this study.

We showed that serum leptin levels increased in the obese group and were correlated with BMI and HOMR-IR. These findings are already well known [22], but it is meaningful for clinical purposes to confirm these results in modestly obese subjects. Nevertheless, unlike previous studies, we were unable to find differences between the insulin-resistant group and the insulin-sensitive group of obese patients in terms of serum leptin levels [26, 27]. We suggest that the reasons for this discrepancy may be related to differences in the study population and the role of adiponectin. In fact, adiponectin may be a more responsive hormone than leptin, especially in patients with modest-obesity-induced insulin resistance.

### Infiltration of immune cells and expression of MCP-1 and TNF-α in VAT

Prolonged caloric overload leads to adipose expansion with adipocyte hypertrophy. As a result, there is a secretion of chemoattractants, such as MCP-1, and proinflammatory cytokines, such as TNF-α, IL-1, and IL-6, causing infiltration of the immune cells [28]. Increased secretion of proinflammatory cytokines, including MCP-1 and TNF-a, induces additional macrophage recruitment and adipocyte dysfunction [8, 29]. These serial inflammatory changes in AT induce a chronic state of inflammation strongly implicated in the mechanisms underpinning whole-body metabolic dysregulation.

This study found a significant increase in the MCP-1 mRNA and TNF-α mRNA expression levels in the VAT of the modestly obese group. However, we found no inter-group differences in the infiltration of macrophages or VAT inflammatory scores between groups according to BMI or glucose tolerance. This observation is not in agreement with previous studies that found that increased infiltration of macrophages was associated with insulin resistance in obese patients [30, 31]. Our results show that an increase in proinflammatory cytokines, such as MCP-1 and TNF-α, in VAT precedes the infiltration of immune cells during the progression of AT inflammation in obese individuals.

TNF-α is highly overexpressed in the AT of obese humans and rodents, and their blockade leads to increases in insulin sensitivity [32–34]. MCP-1 is one of the novel inflammatory biomarkers in obese patients, and it is upregulated in genetic obesity and diet-induced obesity, but only a few studies have addressed the expression of MCP-1 in the VAT of obese humans [12, 35, 36]. One study revealed that MCP-1 transcripts in human VAT were correlated with adiposity and weight loss reduced MCP-1 levels in a severely obese group [36]. In this study, the expression of MCP-1 mRNA was correlated with BMI but not with HOMA-IR, and the expression of TNF-α mRNA was correlated with HOMA-IR. These results support the facts that expansion of AT induces the secretion of MCP-1 and that increased proinflammatory cytokines cause insulin resistance.

### Expression of macrophage markers and mitochondria-associated genes

Many studies have demonstrated that ATMs are responsible for increased proinflammatory cytokines and may contribute to obesity-associated inflammation, insulin resistance, and metabolic dysfunction [37]. Within lean mice, macrophages predominantly exhibited a more M2-like phenotype, expressing CD206, CD301, and arginase 1, along with secretion of anti-inflammatory cytokines [38]. During the early stages of mild obesity in mice fed a high-fat diet (HFD), obesity not only stimulates infiltration of M1 macrophages, which gives rise to the proinflammatory environment, but also alters secretion of chemokines and cytokines such as TNF-α and IL-6. In severe obesity with insulin resistance, the ATM phenotypic switch toward the proinflammatory M1 state overwhelms the protective effects of M2 macrophages [31, 39, 40]. Recently, several studies have investigated the role of macrophages in human adipose tissue inflammation [8, 9, 41] and observed significant changes in the degree of macrophage infiltration in severe human obesity. These results suggest that macrophages play an important role in adipose tissue inflammation, and that this increase in the number of macrophages present in obese human visceral WAT is responsible for the enhanced production of chemokines in this population. However, despite these observations, human studies have yielded contradictory results, with no definite M2 marker identified to date [39]. To address these inconsistencies, we sought to examine the rate of macrophage infiltration and ATM phenotype switching in modestly obese humans. No differences in CD163 or CD206 expression levels, AT inflammation, or crown-like structures were observed between the obese and lean groups, indicating that neither increased macrophage infiltration nor phenotypic switching occurred in modestly obese humans, despite evidence of an insulin resistance-like phenotype. However, genetic expression was investigated in only VAT, and this factor may not always reflect the protein level. Thus, these results should be confirmed using fluorescence-activated cell sorting (FACS) analysis. In addition, many studies suggest that it is impossible to distinguish recently recruited cells from the resident pool of macrophages and to unequivocally identify M1 or M2 polarization. This is the greatest limitation for a human ATM study [42].

To determine which factor is most strongly related to macrophage phenotype in patients with modest obesity, we analyzed the relationships of macrophage markers with metabolic parameters and inflammatory biomolecules. The CD163/CD68 expression ratio was significantly associated with genes involved in mitochondrial biogenesis and OxPhos, such as PGC-1α, PGC1-β, and NDUFA. Although alternative activation of macrophages for fatty acid oxidation and mitochondria biogenesis by PGC-1β may be related to obesity [43–45], the relationship of PGC-1α and the OxPhos complex to macrophages has not yet been established in humans. Based on this study, the regulation of M2 macrophages in patients with modest obesity may be closely linked to mitochondrial biogenesis through PGC-1α as well as PGC-1β, although further research is required to confirm this hypothesis.

Serum resistin levels and leptin levels were significantly correlated with CD163/CD68 expression. Human resistin is primarily expressed in macrophages, and its expression in human AT induces the production of inflammatory cytokines in obesity [46, 47]. Recent human studies have demonstrated that leptin contributes to increased oxidative stress in macrophages and stimulates the proliferation and activation of circulating monocytes [48, 49]. Another study showed that leptin induces M2 polarization [50]. However, the roles of resistin and leptin in the regulation of macrophages are not yet clear. Although we could not identify the underlying mechanism or the link among resistin, leptin, and CD163/CD68 expression levels, our data suggest that serum resistin levels and leptin levels have specific roles in the regulation of ATM in patients with modest obesity.

The most significant limitations of this study included the low number of enrolled patients and its cross-sectional design without intervention. Furthermore, mRNA levels were checked without using FACS analysis.

## Conclusions

In conclusion, these results suggest that changes in the production of inflammatory biomolecules precede increases in immune cell infiltration and macrophage phenotype switching in VAT. Furthermore, serum resistin and leptin have specific roles in the regulation of ATM in patients with modest obesity and early metabolic dysfunction. Further studies examining a larger patient population, as well as direct intervention studies, will be necessary to fully characterize changes in inflammatory cell populations.

## Supporting Information

S1 Table Primer pairs used for mRNA determinatation. (DOCX)

S2 Table Characteristics of the study population in relation to glucose tolerance. (DOCX)

S3 Table Inflammatory markers of the study population in relation to glucose tolerance. (DOCX)
