# Supplementary material for: The Roles of Adipokines, Proinflammatory Cytokines, and Adipose Tissue Macrophages in Obesity-Associated Insulin Resistance in Modest Obesity and Early Metabolic Dysfunction
Source: PLoS One. 2016 Apr 21;11(4):e0154003. doi: 10.1371/journal.pone.0154003 (PMC4839620; doi:10.1371/journal.pone.0154003)
Supplement: S1 Table — (DOCX) [file pone.0154003.s001.docx]

Supporting Table 1. Primer pairs used for mRNA determinatation

|  | Sense primer | Antisense primer |
| --- | --- | --- |
| adiponectin | AGG GAG ACA TCG GTG AAA CC | GTT CTC CTT TCC TGC CTT GG |
| MCP-1 | ACC ATT GTG GCC AAG GAG AT | GTC CAT GGA ATC CTG AAC CC |
| TNF-α | TCT TCT GCC TGC TGC ACT TT | GGC CAG AGG GCT GAT TAG AG |
| CD68 | TCA GCT TTG GAT TCA TGC AG | TTG TAC TCC ACC GCC ATG TA |
| CD163 | TTG CCA GCA GTT AAA TGT G | AGG ACA GTG TTT GGG ACT GG |
| CD206 | CGA GGA AGA GGT TCG GTT CAC C | GCA ATC CCG GTT CTC ATG GC |
| PGC-1 α | CCT TGC AGC ACA AGA AAA CA | TGA CCG AAG TGC TTG TTC AG |
| PGC-1 β | CCG AGC TCT TCC AGA TTG AC | CGA AGC TGA GGT GCA TGA TA |
| NDUFA | CGA GAC TGG GAA ACC AAA AA | GCT TCC TTG GAC AGT TGA GC |
| 18S | AGGAATTCCCAGTAAGTGCG | GCCTCACTAAACCATCCAA |
