# Supplementary material for: The Roles of Adipokines, Proinflammatory Cytokines, and Adipose Tissue Macrophages in Obesity-Associated Insulin Resistance in Modest Obesity and Early Metabolic Dysfunction
Source: PLoS One. 2016 Apr 21;11(4):e0154003. doi: 10.1371/journal.pone.0154003 (PMC4839620; doi:10.1371/journal.pone.0154003)
Supplement: S2 Table — (DOCX) [file pone.0154003.s002.docx]

Supporting Table 2. Characteristics of the study population in relation to glucose tolerance

| Variables | NGT  (N=25) | Prediabetes  (N=17) | T2DM  (N=9) | *P* value |
| --- | --- | --- | --- | --- |
| Age (years) | 46.4±6.91 | 47.71±4.10 | 44.78±8.53 | 0.543 |
| BMI (Kg/m^2^) | 23.80±2.54 | 24.43±2.24 | 25.32±4.88 | 0.411 |
| Waist circumference (cm) | 80.45±5.81 | 81.29±5.09 | 86.52±12.70 | 0.102 |
| Body fat (%) | 29.94±4.63 | 29.90±4.45 | 32.90±7.49 | 0.302 |
| Body fat mass (Kg) | 17.91±4.01 | 18.47±4.63 | 24.18±9.60 | 0.018 |
| Muscle mass (Kg) | 37.91 ± 4.80 | 62.64 ± 89.78 | 40.09 ± 7.20 | 0.278 |
| Fasting glucose (mg/dL) | 94.04±4.95 | 98.00±10.09 | 152.22±53.13 | 0.000 |
| Fasting insulin (mIU/L) | 8.77±3.77 | 9.18±3.33 | 8.57±4.09 | 0.907 |
| Postload 2hrs glucose (mg/dL) | 115.25±15.94 | 163.53±20.57 | 327.22±97.92 | 0.000 |
| Postload 2hr insulin (mIU/L) | 40.49±26.15 | 76.88±48.74 | 65.07±58.91 | 0.027 |
| HbA1c (%) | 5.14±0.33 | 5.37±0.43 | 7.6±1.63 | 0.000 |
| HOMA-IR | 2.00±0.89 | 2.26±0.98 | 3.38±2.37 | 0.027 |
| HOMA-β | 110.20 ± 45.63 | 94.90 ± 32.37 | 44.62 ± 33.10 | 0.012 |
| TG (mg/dL) | 94.08±55.50 | 129.53±93.48 | 125.67±63.63 | 0.244 |
| LDL-chol (mg/dL) | 111.11±29.58 | 117.21±30.20 | 118.44±48.95 | 0.784 |

Data are presented as mean ± SD

*P* value from ANOVA for continours parametric variables and Kruskal-Wallis test for nonparametric variables.
