# Supplementary material for: The Roles of Adipokines, Proinflammatory Cytokines, and Adipose Tissue Macrophages in Obesity-Associated Insulin Resistance in Modest Obesity and Early Metabolic Dysfunction
Source: PLoS One. 2016 Apr 21;11(4):e0154003. doi: 10.1371/journal.pone.0154003 (PMC4839620; doi:10.1371/journal.pone.0154003)
Supplement: S3 Table — (DOCX) [file pone.0154003.s003.docx]

Supporting Table 3. Inflammatory markers of the study population in relation to glucose tolerance

| Variables | NGT  (N=25) | Prediabetes  (N=17) | DM  (N=9) | *P* value |
| --- | --- | --- | --- | --- |
| *Serum* |  |  |  |  |
| Adiponectin (μg/mL) | 14.82±14.04 | 10.76±8.00 | 7.14±6.12 | 0.188 |
| Leptin (ng/mL) | 8.92±7.04 | 9.23±3.90 | 11.14±11.63 | 0.727 |
| Resistin (ng/mL) | 47.12±28.51 | 61.16±38.10 | 35.50±28.59 | 0.385 |
| IL 1β (pg/mL) | 4.46±9.38 | 1.41±1.89 | 1.21±0.94 | 0.266 |
| MCP-1 (pg/mL) | 210.12±91.81 | 189.49±101.57 | 194.11±74.00 | 0.759 |
| TNF-α (pg/mL) | 4.15±2.53 | 4.99±3.38 | 4.58±3.65 | 0.676 |
| hs CRP (mg/L) | 0.78±0.76 | 0.83±0.65 | 0.93±0.71 | 0.869 |
| *Visceral adipose tissue (mRNA expression/18s)* |  |  |  |  |
| Adiponectin | 0.29±0.37 | 0.37±0.38 | 2.00±3.43 | 0.656 |
| MCP-1 | 16.54±15.23 | 6.60±10.21 | 9.15±17.25 | 0.208 |
| TNF-α | 338.58±794.43 | 186.95±372.35 | 69.40±80.77 | 0.938 |
| CD68 | 42.18±180.92 | 7.51±12.99 | 12.79±18.85 | 0.669 |
| CD163/CD68 | 3.11±3.35 | 11.27±14.62 | 6.31±7.27 | 0.116 |
| CD206/CD68 | 12.54±23.57 | 8.93±16.90 | 10.21±25.60 | 0.673 |

Data are presented as mean ± SD

*P* value from ANOVA for continours parametric variables and Kruskal-Wallis test for nonparametric variables.
